# Small-angle X-ray scattering: characterization of cubic Au nanoparticles using Debye’s scattering formula

**DOI:** 10.1107/S160057672200499X

**Published:** 2022-07-15

**Authors:** Jérôme Deumer, Brian R. Pauw, Sylvie Marguet, Dieter Skroblin, Olivier Taché, Michael Krumrey, Christian Gollwitzer

**Affiliations:** a Physikalisch-Technische Bundesanstalt (PTB), Abbestraße 2–12, 10587 Berlin, Germany; b Federal Institute for Materials Research and Testing (BAM), Unter den Eichen 87, 12205 Berlin, Germany; c Université Paris-Saclay, CEA, CNRS, NIMBE, 91191 Gif-sur-Yvette, France; DESY, Hamburg, Germany

**Keywords:** small-angle X-ray scattering, non-spherical nanoparticles, Debye scattering equation

## Abstract

A versatile software package in the form of a Python extension, named *CDEF* (computing Debye’s scattering formula for extraordinary form factors), is proposed to calculate approximate scattering profiles of arbitrarily shaped nanoparticles for small-angle X-ray scattering (SAXS). *CDEF* generates a quasi-randomly distributed point cloud in the desired particle shape and then applies the open-source software *DEBYER* for efficient evaluation of Debye’s scattering formula to calculate the SAXS pattern (https://github.com/j-from-b/CDEF). If self-correlation of the scattering signal is not omitted, the quasi-random distribution provides faster convergence compared with a true-random distribution of the scatterers, especially at higher momentum transfer. The usage of the software is demonstrated for the evaluation of scattering data of Au nanocubes with rounded edges, which were measured at the four-crystal monochromator beamline of PTB at the synchrotron radiation facility BESSY II in Berlin. The implementation is fast enough to run on a single desktop computer and perform model fits within minutes. The accuracy of the method was analyzed by comparison with analytically known form factors and verified with another implementation, the *SPONGE*, based on a similar principle with fewer approximations. Additionally, the *SPONGE* coupled to *McSAS3* allows one to retrieve information on the uncertainty of the size distribution using a Monte Carlo uncertainty estimation algorithm.

## Abstract 2

The Python extension *CDEF* is a suitable evaluation tool for experimentalists to calculate single-particle small-angle X-ray scattering profiles with satisfactory accuracy, as shown by comparison with common well-known analytical form factors. Using different complex cubic models, a direct comparison between *CDEF* and the already established *SPONGE* with respect to the size distribution of imperfect Au nanocubes showed a clear agreement of the results.

## 1. Introduction

Small-angle X-ray scattering (SAXS) is a powerful nano­structure quantification tool to characterize ensembles of nanoparticles (Guinier & Fournet, 1955 ▸). The X-ray scattering pattern of a nanoparticle system depends on many particle properties, which can therefore be obtained from the measurement, such as the radius of gyration (Guinier & Fournet, 1955 ▸), particle shape (Guinier, 1939 ▸; Guinier & Fournet, 1955 ▸; Porod & Glatter, 1982 ▸), size distribution (Riseman, 1952 ▸), specific surface area (Guinier & Fournet, 1955 ▸) and number concentration (Schavkan *et al.*, 2019 ▸).

It is a nondestructive method with only little sample preparation for particles in liquid suspension and is also applicable for powders and porous materials (Bock *et al.*, 1997 ▸). With SAXS, typically particles with sizes ranging from a few nanometres up to a few hundred nanometres can be measured if there is sufficient electron density contrast of the particles relative to the suspension medium, since photons are scattered by the electrons in the material. The higher the electron density contrast, the more pronounced the scattered intensity relative to the background signal originating from the suspension. The measured SAXS signal can be further processed and fitted to obtain information about the desired particle properties.

To fit and evaluate experimental data, an adequate assumption of the underlying particle shape is necessary. This assumption is made by choosing the correct form factor *F*(*q*) for the physical model, where *q* is the magnitude of the scattered photon’s momentum transfer vector.

For simple particle shapes such as spheres, cylinders or spherical core–shell particles, *F*(*q*) can be calculated analytically. For instance, *F*(*q*) of a perfect sphere with a homogeneous electron contrast Δρ was derived by Rayleigh (1911 ▸):

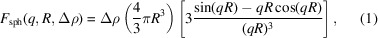

where *R* is the radius of the sphere.

The scattering pattern *I*(*q*) of a polydisperse particle ensemble, as measured on the detector, is then obtained by convolving the absolute square of the form factor |*F*_sph_(*q*, *R*, Δρ)|^2^ with the size distribution *g*(*R*): 

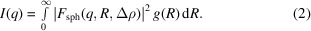

Equation (1) can be extended to other geometrical shapes with spherical symmetry, such as core–shell particles and particles with multiple concentric shells (Pedersen, 2002 ▸; Kohlbrecher, 2020 ▸). For regular shapes with lower symmetry, the form factors are known, among many others, for ellipsoids (Guinier, 1939 ▸), cylinders (Guinier & Fournet, 1955 ▸), cubic particles (Mittelbach & Porod, 1961 ▸), and cylindrical and conical particles with an arbitrary polygonal base which are built out of polygonal wedges (Shapovalov, 2013 ▸). For all these shapes, the average over all possible particle orientations is typically performed by numerical integration. This requires a one-dimensional average for shapes with one axis of rotational symmetry, such as cylinders and ellipsoids, and a two-dimensional average for others like cubic shapes (Mittelbach & Porod, 1961 ▸; Napper & Ottewill, 1963 ▸; Pedersen, 2002 ▸; Nayuk & Huber, 2012 ▸), which is costly.

Recently, a seemingly limitless landscape of nanomaterial shapes and structures that do not fit these analytical functions have been synthesized, such as stars (Zhou *et al.*, 2015 ▸; Feld *et al.*, 2019 ▸), cubes with concave faces (Zhou *et al.*, 2015 ▸) and core–shell-structured cubes (Zhou *et al.*, 2015 ▸; Jia *et al.*, 2016 ▸; Feld *et al.*, 2019 ▸), demanding a convenient method of calculating scattering profiles *I*(*q*) of these complex-shaped particles. Widely used SAXS analysis software such as *SASfit* (Breßler, Kohlbrecher & Thünemann, 2015 ▸) or *SasView* (https://www.sasview.org/) provides extended libraries of analytic form factors to evaluate SAXS data. However, analytic expressions for a particular shape may not be readily available, and the derivation can quickly become intractable (Shapovalov, 2013 ▸).

A viable alternative approach to the analytic treatment of form factors for irregular shapes consists of building an approximation of the desired shape from smaller objects and calculating the scattering of the approximation via the Debye (1915 ▸) scattering equation, which allows direct computation of the rotational average of an ensemble of scatterers from their individual form factors. Hansen (1990 ▸) has proposed to build irregular shapes from randomly distributed point scatterers, and Pedersen *et al.* (2012 ▸) successfully applied this method to the analysis of polydisperse immune stimulating complex vaccine particles, which are perforated bilayer vesicles with or without proteins, composed of compounds with different scattering length densities.

With the present paper, we introduce our open-source software *CDEF* (Deumer & Gollwitzer, 2022 ▸), which provides efficient calculation of approximate scattering profiles *I*(*q*) for polydisperse ensembles of arbitrarily shaped nanoparticles. *CDEF* builds on the ideas put forward by Hansen (1990 ▸) and Pedersen *et al.* (2012 ▸) and enhances them with the option for quasi-random distribution of scatterers, which can improve convergence. An additional speed-up is achieved by offloading the actual calculation of the Debye formula to the open-source software *DEBYER* (Wojdyr, 2020 ▸).

The algorithm is detailed in Section 2. As an application, *CDEF* is used to evaluate scattering data from gold nanocubes with rounded edges in Section 3. Experimental details and the used nanomaterial are described in Sections 4 and 3, respectively. Finally, the results are compared with the pre-existing program the *SPONGE*, based on similar principles (Aratsu *et al.*, 2020 ▸), in Section 5.

## 2. Methods

In this section, *CDEF* and the *SPONGE* will be described in more detail. Both programs are based on the Debye (1915 ▸) scattering formula, which can generally be used to calculate the SAXS pattern *I*(*q*) of a system of *N* individual scatterers, 

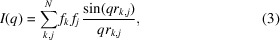

from the form factors *f*_*i*_ of the individual scatterers and the distances *r*_*k*,*j*_ between the scatterers *k* and *j*.

### 2.1. Implementation details of *CDEF*

**Figure 1:**
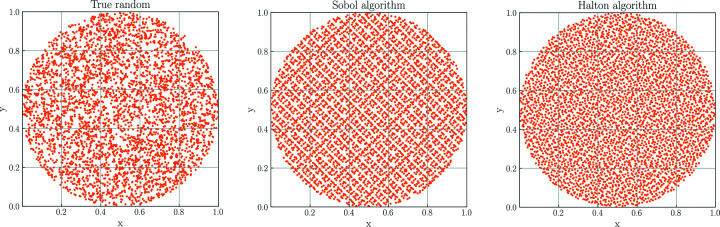
Examples of circular point clouds with radius *r* = 0.5 generated with one true-random and two quasi-random (Sobol, Halton) filling algorithms. Each cloud is generated by initially filling 5000 points into a squared area with side length *l* = 1 and subsequently deleting all points outside of the circle, which leaves ∼4000 points. The usage of a quasi-random algorithm leads to a higher homogeneity of the spatial distribution relative to the true-random method, whereas the true-random distribution shows a higher degree of local clustering.

To approximately calculate *I*(*q*) for arbitrarily shaped nano­particles, *CDEF* applies equation (3) to a three-dimensional point cloud of the desired particle shape. The point cloud is created by filling the particle’s bounding box with equally distributed punctiform scatterers and discarding all points outside of the volume defined by the particle’s shape. The shape can be built either from a computer-aided design (CAD) construction (for this, both *CDEF* and the *SPONGE* offer the import of the widely used STL file format) or by programmatically reshaping the point cloud. *CDEF* provides the option to generate the initial point cloud from either a true- or a quasi-random sequence (Fig. 1 ▸). Compared with the true-random series, a quasi-random sequence fills the shape more evenly with less local clustering (Vandewoestyne & Cools, 2006 ▸). We implemented a generator for the scrambled Halton (1964 ▸) series proposed by Kocis & Whiten (1997 ▸) and Sobol’s (1967 ▸) series as provided by the *SciPy* package (Virtanen *et al.*, 2020 ▸).

Each point of the generated cloud then gets assigned a weight to account for density variations such as in heterogeneous or core–shell particles. Finally, as a computational tool to efficiently evaluate Debye’s scattering formula, *CDEF* passes the points and the associated weights to the open-source program *DEBYER* (Wojdyr, 2020 ▸), whereas the *SPONGE* uses its own implementation of the Debye equation. Similar approaches to compute form factors for arbitrary shapes using Debye’s scattering formula have been reported by Pedersen (2002 ▸), Pedersen *et al.* (2012 ▸) and Hansen (1990 ▸), and are used by other fast programs, *e.g.* *DEBUSSY* (Cervellino *et al.*, 2015 ▸).

A more detailed comparison between *CDEF*, the *SPONGE* and other evaluation methods using Debye’s equation can be found in the supporting information (SI).

As proposed by Hansen (1990 ▸) and Pedersen *et al.* (2012 ▸), *DEBYER* achieves a significant performance gain by splitting the calculation of equation (3) into two parts. First a histogram of the pair distances *r*_*k*,*j*_ is computed with a reduced number of histogram bins *N*_BINS_, and subsequently the sinc function 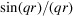 is evaluated for each bin of the histogram. Because *N*_BINS_ is usually much smaller, typically around 1000–10 000, than the number of pairs of scatterers *N* ^2^, this approximation can speed up the computation by several orders of magnitude for repeated evaluation of equation (3) for different *q*, such as in the computation of a full scattering pattern. *CDEF* allows the user to set the histogram bin width explicitly to trade off the accuracy of the computed scattering curve with computation time.

The scattering pattern *I*_MONO_ obtained in this way corresponds to a single particle, averaged over all possible orientations. For the modeling of realistic particle dispersions, *I*_MONO_ must be averaged over a certain size distribution. *CDEF* achieves this by rescaling the single-particle scattering curve from a single master curve according to 

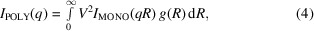

which avoids repeated evaluation of Debye’s scattering equation for different particle sizes. Here, *g*(*R*) is the size distribution and *V* the volume of the rescaled particle with size *R*. The integral in equation (4) is evaluated by Monte Carlo integration with 3000 samples using a normal random-number generator, yielding a Gaussian size distribution, but other distributions can be easily implemented by using the appropriate random-number generator. At the moment, *CDEF* implements Gaussian and lognormal distributions.

The implementation of a Poisson disc algorithm to fill the bounding box homogeneously with scatterers which are required to have a certain minimum distance to each other would also be conceivable. However, this requires more computational effort, *e.g.* filling a cube with 30 000 points is approximately 28 times slower [∼350 ms (Sobol) versus 9.85 s], and would not offer any apparent advantages over the existing algorithms (SI).

### 2.2. *CDEF* versus analytic formulae

**Figure 2:**
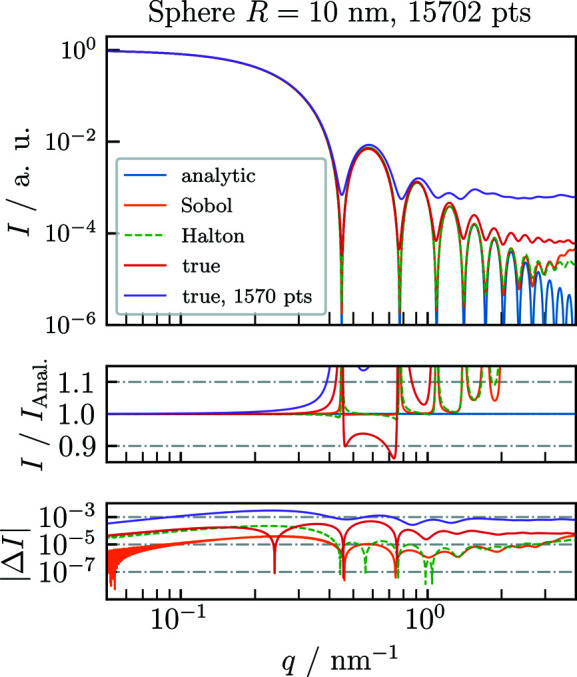
Comparison of normalized single-particle SAXS profiles, obtained using *CDEF* without modeling of the artificial background signal, with the exact analytic SAXS profile *I*_Anal._ of a sphere with radius *R* = 10 nm and electron contrast Δρ = 1 nm^−3^. For the numeric calculations, the Deybe equation was applied on spherical clouds which were generated using two different quasi-random (Sobol, Halton) and one true-random filling algorithm. At specific *q* values the artificial scattering signal from the fine structure of the individual cloud dominates the numeric profiles, leading to a deviation from *I*_Anal._.

As a validation of *CDEF*, we first compare its normalized results with the corresponding analytic form factors of common particle shapes using the three introduced filling algorithms (Fig. 1 ▸). Fig. 2 ▸ shows the analytically [equation (1)] and numerically calculated single-particle SAXS profiles of a homogeneous sphere with radius *R* = 10 nm. For the calculation of each numeric profile, a spherical cloud was generated by (quasi-)randomly filling 30 000 points into a cubic bounding box with side length 2*R* = 20 nm and then deleting all points outside of the defined sphere, which yields *N* ≃ 15 700 remaining points.

Both quasi-random profiles match the analytic profile with good agreement up to the fifth local maximum, whereas at higher *q* values both profiles start deviating from the analytic profile owing to an artificial background signal originating from the clouds’ fine structure. This also holds true for the true-random filling pattern with the same number of scattering points. However, it only matches *I*_Anal._ up to the second local maximum because of the constant scattering background. Fig. 2 ▸ also shows that reducing the number of scattering points by a factor of 10 raises the background plateau by the same factor.

Pedersen *et al.* (2012 ▸) proposed that the constant background arising from the true-random distribution can be subtracted by excluding the self-correlation of the scatterers, which corresponds to zeroing the first bin of the pair distance histogram or subtraction of a constant value of 1/*N* from the resulting scattering patterns. This does indeed increase the dynamic range of the computed scattering curve and brings it into closer agreement with the true pattern.

**Figure 3:**
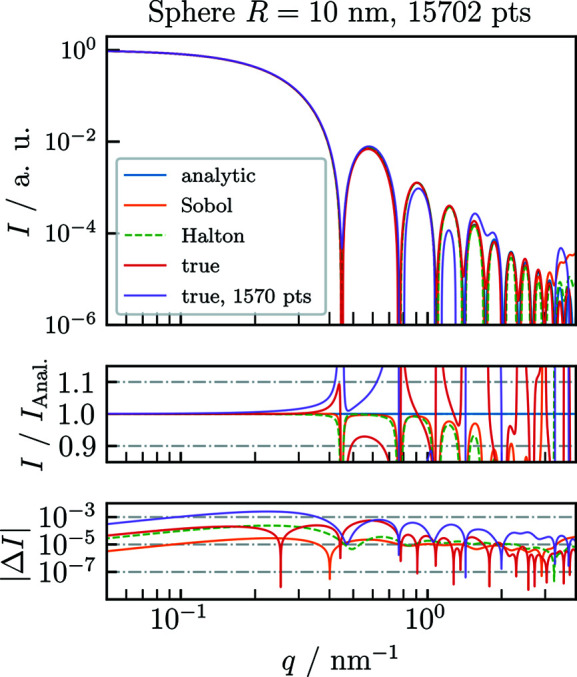
Comparison of normalized single-particle SAXS profiles with modeling of the artificial background signal.

Fig. 3 ▸ displays background-corrected scattering patterns for the three different types of filling algorithm. For the quasi-random filling algorithms, zeroing the first bin does not improve the agreement with the exact solution because of the low autocorrelation at small distances of quasi-random sequences. Instead, zeroing a small initial sequence of bins except for the first can bring the curves into closer agreement with the exact result (see Fig. 3 ▸). Still, the curve computed from the quasi-random sequences without this correction is in better agreement for midrange values of *q* than the corrected true-random solution, which is evident by comparing the plots of the relative deviation in Figs. 2 ▸ and 3 ▸. To perform these optimizations for a given case, *CDEF* provides the option to zero out a sequence of bins in the pair distance distribution histogram.

Similar results are obtained for a comparison of particles with lower symmetry, such as cylinders and cubes. The corresponding data can be found in the SI (Figs. S2, S5, S6 and S7).

### 2.3. The *SPONGE*

#### 2.3.1. Implementation details of the *SPONGE*

A separate implementation was developed, called the *SPONGE* (Pauw & Breßler, 2022 ▸), that is a more fundamentally proximate method by eschewing many of the speed-improving approximations. It also uses the Debye equation for puctiform scatterers with a fully random point distribution. This method is essentially similar to *CDEF* with the exception that the intermediate step, where the numerical pair distance distribution function is generated, is bypassed in favor of a more direct approach, further minimizing potential sources of error.

While the *SPONGE* is much more computationally intensive, it should be more accurate over the entire *q* range where the homogeneous phase approximation holds, and thus it can be used to validate that the approximations in the faster *CDEF* implementations are not generating unforeseen artifacts. Like *CDEF*, the *SPONGE* uses a surface description in the STL format to define the boundaries of a nano-object. It then leverages the fast VTK bindings in Python (Schroeder *et al.*, 2006 ▸) for point placement and determines whether the point lies inside or outside of the object. The computation of the point-to-point Euclidian distance matrix is done using a fast *SciPy* implementation (The SciPy Community, 2021 ▸), before the Debye equation is applied to obtain a simulated isotropic scattering curve. When a scattering length density is provided, the *SPONGE*-simulated data can be scaled to absolute units (*i.e.* to an absolute scattering cross section in m^−1^ sr^−1^).

This procedure is repeated, resulting in a number of independently generated scattering curves, each based on their own set of random points. The mean intensity from all repetitions is then presented, with the standard deviation used as an estimate for the uncertainty for each point.

A number-weighted size distribution can also be taken into account. The *SPONGE* currently uses a Gaussian size distribution, which is implemented by choosing a random scaling factor for the *q* value for each independent repetition and which affects the total intensity scaling factor according to its scaled volume (in a procedure identical to that given in Section 2.1). This would be similar in reality to probing a multitude of objects of different size to build up the average scattering pattern. This size distribution has been verified to work accurately (by checking the result with a fit in *SasView*) up to a Gaussian distribution width σ of at least 50%. This simulated distribution width is not used for fitting but is used to avoid unrealistically sharp minima in the simulated curve. For the simulations presented herein, the distribution width is set to 1%.

#### 2.3.2. The *SPONGE* and *McSAS3*

The thus-simulated data of primary particles can be used to fit an experimental data set, even when the experimental data set is from a sample with an unknown, broader distribution of particle sizes. For this, we turn the simulated data into a fitting model for use with the Monte Carlo approach as implemented in *McSAS* (Pauw *et al.*, 2013 ▸; Bressler, Pauw & Thünemann, 2015 ▸). As the original *McSAS* is not easily adapted to support such a model description, we are here using the refactored *McSAS3* implementation (currently in the last stages of development). *McSAS3* works using the same methods as *McSAS* but has many practical improvements, such as multi-threaded optimization, a backend independent of the graphical user interface (for headless computation) and the option to re-histogram a previous optimization run (*McSAS* on GitHub; Breßler & Pauw, 2022 ▸).

The simulated data set can be converted into a fitting model, provided it has a Guinier region at low *q* and (on average) a Porod region at high *q*. Then, for a given scaling factor, the *q* value of the simulated data is rescaled (in a manner identical to Section 2.3.1), and the intensity interpolated to the requested *q* value of the experimental data. Data points that fall outside the limits of the simulated data are extrapolated using a flat (Guinier) approximation at low *q* and a Porod slope at high *q*.

Using this fitting model in *McSAS3*, experimental data can be fitted rapidly using the simulated scattering pattern of an elementary scatterer. From this, a form-free volume-weighted scaling factor distribution is obtained that best describes the experimental data. As with the original *McSAS* (Pauw *et al.*, 2013 ▸), a number of independent optimizations are performed to allow the estimation of the uncertainty of the resulting distribution.

### 2.4. Diverse models of cubic particles

**Figure 4:**
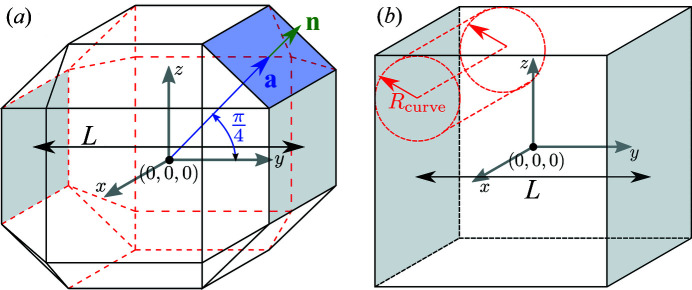
Two different cubic models with face-to-face-distance *L*. (*a*) Cube with truncated edges. All 12 edges are truncated by sectional planes. Each sectional plane (an example is marked in blue) is defined by a support vector **a** and a normal vector **n** which stands perpendicular to the plane. (*b*) Cube with implied rounded edges. The curve of each edge is defined by identical cylinders with curvature radius *R*_curve_ which touch the associated cubic sides.

To show the versatile application of *CDEF*, we characterize the Au nanocubes that are described in Section 3. In doing so, we implemented three different cubic models (ideal cube, cube with truncated edges, cube with rounded edges) carrying a homogeneous electron density (Fig. 4 ▸).

To simulate truncated edges, an advanced (*i.e.* point clouds are generated by user-written Python functions) algorithm based on the Hessian normal form is implemented, with which the truncation level of the cubic model with a side-to-side distance *L* can be adjusted. Further information is provided in the SI.

Moreover, a cubic model with rounded edges is generated by introducing four cylinders for each Euclidean direction *x*, *y*, *z*, where each cylinder is located in one of the four corners with its axis being aligned along the corresponding edge (Fig. 4 ▸). The rounded edges are then generated by deleting points, *i.e.* setting their corresponding form factor to zero, located at the edges and outside of each cylinder. All 12 cylinders are described by the same radius of curvature *R*_curve_.

## 3. Synthesis of Au nanocubes

**Figure 5:**
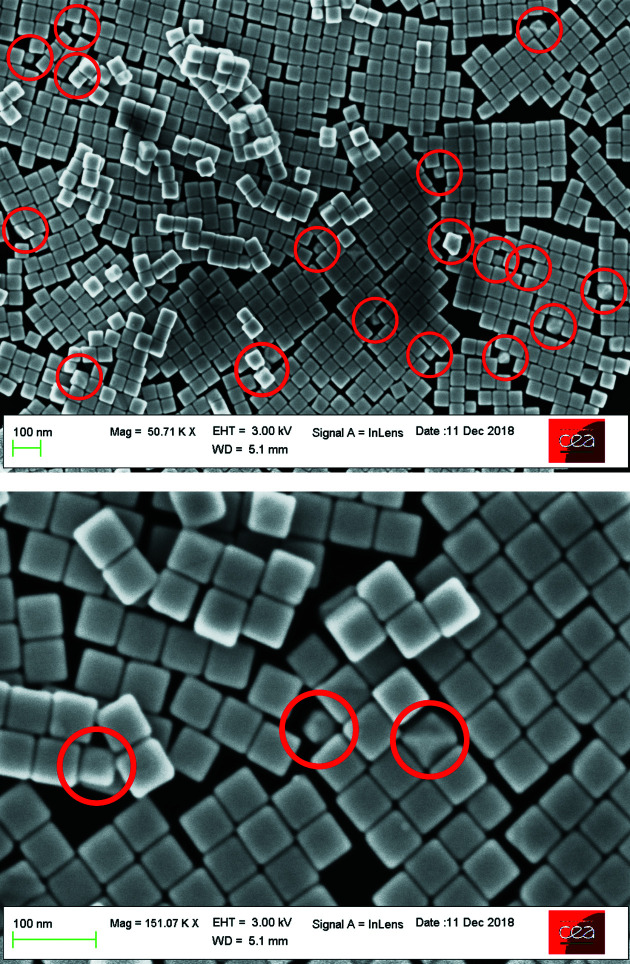
Scanning electron microscopy images of Au nanocubes at two different scales. The population also consists of a few particles with a non-cubic shape (some marked by red circles).

Mono-crystalline Au nanocubes (Fig. 5 ▸) were prepared by colloidal chemistry in aqueous solution, according to an already published protocol (Haggui *et al.*, 2012 ▸; Kameche *et al.*, 2020 ▸), in the presence of cetyltrimethylammonium bromide as the capping agent. Crystal growth was achieved by chemical reduction of Au^+^ ions on the surface of a gold seed (a small sphere with an initial size of 2–3 nm in diameter), resulting in the formation of a cubic shape (Kuo *et al.*, 2018 ▸). The side length of these particles as determined from scanning electron microscopy (SEM) images is 55 nm with a standard deviation of 2 nm. Using this particular synthesis procedure leads to a percentage of ∼90% of nanocubes with respect to the whole particle ensemble and a small number (∼10%) of particles with different shapes (see marked spots in Fig. 5 ▸). The edges and corners of the cubes tend to gradually round out over time. In solution, this phenomenon is slow (six months). However, it is faster (one month) when the cubes are deposited on a substrate and kept in air. From the SEM images, a curvature radius (*R*_curve_ ≃ 7 nm) was determined for the edges.

## 4. Experimental details

Since the SAXS experiments were conducted in vacuum, the diluted colloidal solution of Au nanocubes suspended in water was filled into a rectangular capillary of borosilicate glass, with a homogeneous thickness along its vertical axis, and sealed with a blow torch before measurement. The sample was then loaded into the experimental vacuum chamber which is connected to the four-crystal monochromator (FCM) beamline of the PTB laboratory at the synchrotron radiation facility BESSY II, Berlin. For the experiment, X-rays were generated by a bending magnet and then guided by the beamline to the sample holder, resulting in a thin X-ray beam with a cross-sectional area of approximately 150 µm high and 400 µm wide at the sample position. The FCM beamline allows experiments in a wide range of photon energies from *E*_ph_ = 1.75 keV to *E*_ph_ = 10 keV (Krumrey, 1998 ▸). Our SAXS experiments were performed at *E*_ph_ = 8 keV using Si(111) monochromator crystals with a spectral resolving power of *E*_ph_/Δ*E*_ph_ = 10^4^ and a photon flux in the range of Φ ≃ 10^10^ s^−1^ (Krumrey, 1998 ▸). During the experiment, the capillaries were measured at different *y* positions along the vertical axis. At each *y* position, SAXS images were recorded by a vacuum-compatible PILATUS 1M hybrid-pixel detector with a pixel size of *p* = 172 µm (Wernecke *et al.*, 2014 ▸).

### 4.1. Data processing

Prior to data evaluation, the 2D SAXS image, consisting of concentric circles, is converted into the corresponding one-dimensional SAXS profile in absolute units. This allows us to determine the number concentration of suspended particles. For each distinct *y* position, the measured or experimental intensity *I*_EXP_ is circularly integrated around the center of the incident beam and then normalized to the incident photon flux, the duration of exposure, the sample thickness and the quantum efficiency of the detector at a given photon energy (Schavkan *et al.*, 2019 ▸). Then *I*_EXP_ is expressed in terms of the momentum transfer *q*: 

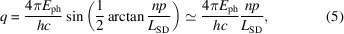

where *L*_SD_ is the distance from the sample to the detector plane, *n* is the number of pixels, *h* is Planck’s constant and *c* is the speed of light. Data processing at PTB, up to this point, is standardized using in-house software.

Since scattering from water molecules and the walls of the glass capillary is also detected by the SAXS measurement, leading to an unwanted background signal, an additional capillary only filled with distilled water was measured during the same measurement to detect the corresponding background curve, which was eventually subtracted from *I*_EXP_. For better statistics, however, *I*_EXP_ and the background curves were averaged over all *y* positions before subtraction.

After subtraction of the background signal, it was not necessary to include an independent background in the fitting model. This also reduces the number of adjustable parameters.

## 5. Results and discussion

In this work, we characterized Au nanocubes using three different cubic models, namely an ideal cube, a cube with truncated edges and a cube with rounded edges. However, for reasons of convenience, only results referring to the model with rounded edges, which shows the lowest χ^2^ (Table 1 ▸), are presented. Detailed results of the other models can be found in the SI.

During the fitting of shapes with varying geometry, such as the truncated or rounded cubic model, it is necessary to recalculate the individual single-particle scattering profile *I*_MONO_ in each computational step. For steady particle shapes with size changes only, such as ideal cubes, it is sufficient to calculate *I*_MONO_ once and then rescale it in accordance to the assumed size distribution, which requires much less computational effort. For all models, no modeling of the artificial background signal was performed, as illustrated in Section 2.2, and a sufficiently high number of scatterers (*N* = 30 000) was chosen to cover the required *q* range.

**Figure 6:**
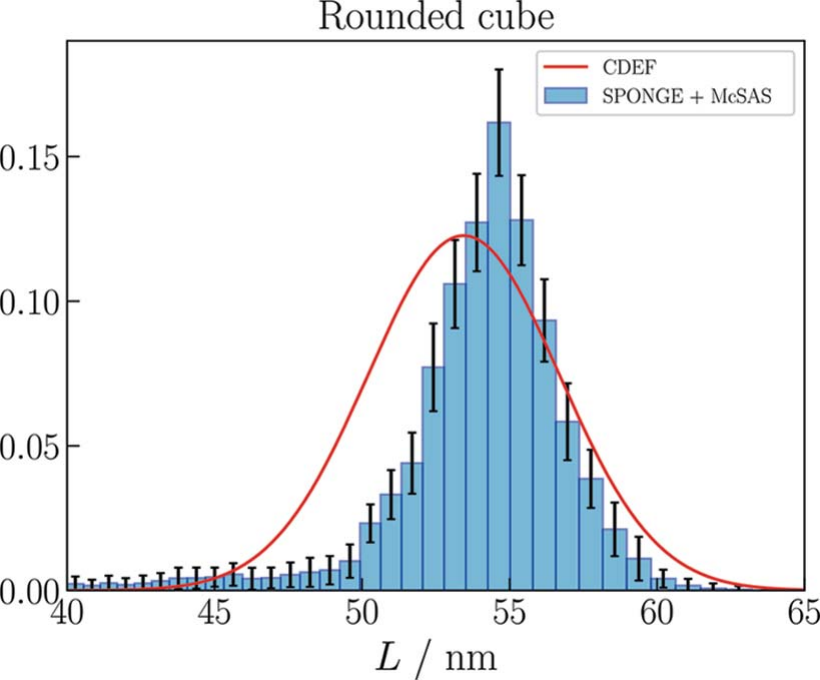
*CDEF* versus the *SPONGE*: the *SPONGE*’s volume-weighted size distribution reveals a mean value of *L* = (54.00 ± 0.06) nm. The volume-weighted distribution using *CDEF* again shows an expectation value of *L* = 53.4 nm.

For all introduced cubic models, the results of the (faster) *CDEF* are compared with those of the *SPONGE* to confirm the results of *CDEF*. Fig. 6 ▸ compares the volume-weighted size distribution from the *SPONGE* with the size distribution from *CDEF*, converted into volume weight. Both methods evaluated the same experimental data. Since the *SPONGE* cannot fit shape parameters owing to the time-consuming computing process, STL files of *CDEF*’s best-fit particle shapes were generated and then given to the *SPONGE* to reveal the underlying uncertainty of *I*_FIT_ (the specific model function or fitting function).

Using *CDEF*, each model was fitted to the experimental data by varying the *M* free parameters, namely the number-weighted distribution of the side-to-side length *L*, which was assumed to be Gaussian, and the truncation or rounding parameters for the imperfect cubes. Powell’s algorithm (Fletcher & Powell, 1963 ▸) with a maximal number of *M* × 1000 function evaluations was used to minimize χ^2^. The combined *SPONGE* + *McSAS* analysis was not confined to any particular size distribution but rather fitted the volume-weighted size distribution numerically.

With *CDEF*, each 3D cloud initially consisted of *N* = 30 000 scattering points. Then for each function evaluation step *N* was varied according to the underlying spatial distribution of scatterers, the level of truncation *T* or the radius of curvature *R*_curve_ such that *N* < 30 000. This initial number of *N* = 30 000 was a good compromise to fit the whole *q* interval of the experimental data without experiencing any artifacts arising from the clouds’ fine structure, but staying below a computing duration of <4 s per evaluation of χ^2^. For comparison, a spherical model was additionally included in the evaluation (Table 1 ▸ and SI).

**Figure 7:**
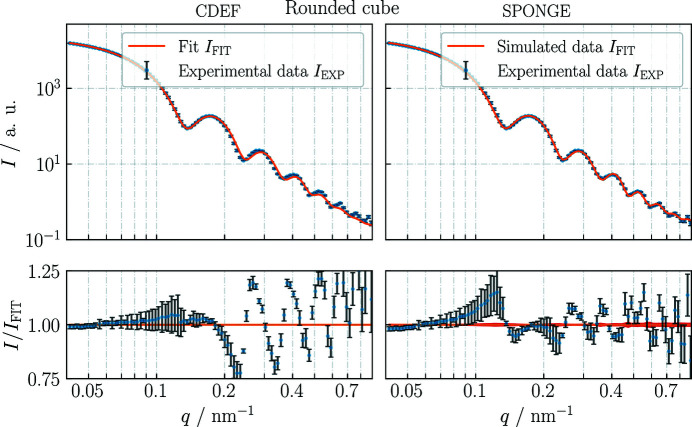
*CDEF* versus the *SPONGE*. Fit results of Au nanocubes using a cubic model with rounded edges. Coupling of the *SPONGE* with *McSAS* additionally reveals an uncertainty of *I*_FIT_, and thus the uncertainty of the underlying size distribution can be stated (Fig. 6 ▸). See Table 1 ▸ for further information.

For both methods, the cubic models with truncated (SI) or rounded edges (Fig. 7 ▸) fit the experimental data slightly better than the ideal cube. The lower values of χ^2^ (Table 1 ▸) also imply a higher degree of compliance for these models, which coincides with the fact that the particles’ edges and corners gradually round out over time when being stored in suspension for more than six months.

For the model with rounded edges we obtain the same result of *L* = 53.4 nm with σ_*L*_ = 3.2 nm. With this model, we additionally obtain a radius of curvature of *R*_curve_ ≃ 7 nm, which is in good agreement with the value measured with SEM (Section 3). With the *SPONGE* we obtain *L* = (54.00 ± 0.06) nm and σ_*L*_ = (3.1 ± 0.9) nm. The relative deviation of the mean face-to-face-distance Δ*L*/*L* again equals 1.1%.

Since the measured ensemble of Au nanocubes does not consist only of cubes with a single shape (ideal, truncated or rounded) but partially contains all of these plus particles with undefined (*i.e.* non-cubic) shapes (Fig. 5 ▸), none of the specific cubic models used is actually able to exactly fit the measurement data, meaning *I*/*I*_FIT_ ≃ 1 and χ^2^ ≤ 1 for the entire *q* range. Also the uncertainty estimate coming from data processing (Section 4.1), meaning the background subtraction in particular, could be underestimated.

Thus, a next step to improve the overall model of the particle ensemble could be the application of a model function including all assumed cubic models with their volume-weighted percentage of the total particle population. The percentage would need to be determined for a representative sample of the ensemble in advance, for instance using microscopic methods with which number-weighted percentages would be obtained.

## 6. Conclusion

*CDEF* is suitable for calculating single-particle SAXS profiles of common particle shapes (including shapes with high aspect ratios) with satisfactory accuracy, which was shown by comparison with known analytic form factors. Here, a sufficient but minimal number of scattering points should be selected to prevent artifacts from appearing in the scattering profile while keeping the computing effort low. Additionally, users of *CDEF* are able to make manual changes to the underlying pair distance histogram to further reduce the number of necessary scattering points. Occasional cross-checks can be made between *CDEF* and the *SPONGE* to ensure that the speed-improving assumptions in *CDEF* are not interfering with the accuracy of the results. Using *CDEF*, polydisperse SAXS patterns can also be generated, eventually allowing experimental data to be evaluated. For all presented cubic models, a direct comparison between *CDEF* and the *SPONGE* concerning the size distribution of Au nanocubes reveals good agreement between results, with a deviation of the mean size of 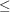 1.5%, even though *CDEF* uses the histogram approximation of the pair distances through *DEBYER* and is confined to a Gaussian distribution.

The time-saving approach of implementing Debye’s equation in *CDEF* further allows us to introduce fit parameters of the particle shape, which enable users to obtain more detailed information on the measured nanoparticles. In terms of ‘steady-shape’ particle evaluation, moreover, *CDEF* has also been coupled with a Markov chain Monte Carlo algorithm to additionally reveal uncertainty estimates of the assumed size distribution of bipyramidal TiO_2_ nanoparticles (Crouzier *et al.*, 2021 ▸).

While the more direct *SPONGE* approach is not quick enough for iterative optimization methods, the coupling of the *SPONGE* with *McSAS3* allows the determination of size distributions of odd-shaped particles when no information on the shape of the analytical size distribution is known. The coupling of *CDEF* with *McSAS3* is, in principle, also possible since both programs are implemented as Python libraries. This would lead to superior performance compared with the *SPONGE* and will be considered in future versions of *CDEF*.

Both approaches can be extended to include core–shell morphologies by varying the density of scatterers or assigning different electron densities to the individual punctiform scatterers. Further speed-up could be achieved by an implementation which runs on parallel hardware such as consumer graphics cards.

## 7. Related literature

The following additional references are cited in the supporting information: Bertolotti *et al.* (2016 ▸), Franke *et al.* (2017 ▸), Galantini *et al.* (2004 ▸), Pauling (1947 ▸), Svergun (1999 ▸) and Svergun *et al.* (1995 ▸).

## Supplementary Material

Supporting information file. DOI: 10.1107/S160057672200499X/yr5077sup1.pdf

## Figures and Tables

**Table 1:** *CDEF*: summary of fitting results of homogeneous cubic models with number concentration *C*, mean particle size *L*, standard deviation σ_*L*_, truncation factor *T* (in terms of *L*/2^1/2^), radius of curvature *R*_curve_ (in terms of *L*/2), number of iterations *N*_iter_ of the Powell algorithm, number of function evaluations *N*_fev_ of the χ^2^ function and computing time *t*

Model	*C* † (cm^−3^)	*L* (nm)	σ_*L*_ (nm)	*T*	*R*_curve_	χ^2^	*N*_iter_	*N*_fev_	*t* (s)
Ideal cube	8.709 × 10^9^	52.5	2.8	–	–	<33	5	262	<37
Truncated cube	8.604 × 10^9^	53.4	3.3	0.91	–	<23	5	433	<1339
Rounded cube	8.562 × 10^9^	53.4	3.3	–	0.27 (7.2 nm)	<21	6	493	<1172
Sphere	8.636 × 10^9^	31.7‡	3.4§	–	–	<158	5	274	<39

† Number concentration is based on an effective electron contrast of Δρ ≃ 4077 nm^−3^ of Au particles suspended in H_2_O at 8 keV.

‡ Spherical radius in nanometres.

§ Standard deviation of radius in nanometres.
