# Supplementary material for: Small-angle X-ray scattering: characterization of cubic Au nanoparticles using Debye’s scattering formula
Source: J Appl Crystallogr. 2022 Jul 15;55(Pt 4):993–1001. doi: 10.1107/S160057672200499X (PMC9348877; doi:10.1107/S160057672200499X)
Supplement: Supplementary file 1 [file j-55-00993-sup1.pdf]

Small-Angle X-ray Scattering: Characterization of cubic Au nanoparticles using Debye's scattering formula

Supporting Information

Jérôme Deumer,[a*] Brian R. Pauw,[b*] Sylvie Marguet,[c*] Dieter Skroblin,[a] Olivier Taché,[c] Michael Krumrey[a] and Christian Gollwitzer [a*]

[a]*Physikalisch-Technische Bundesanstalt (PTB), Abbestr. 2-12, 10587 Berlin, Germany,* [b]*Federal Institute for Materials Research and Testing (BAM), Unter den Eichen 87, 12205 Berlin, Germany, and* [c]*Université Paris-Saclay, CEA, CNRS, NIMBE, 91191 Gif-sur-Yvette, France. E-mail: jerome.deumer@ptb.de, Brian.Pauw@bam.de, sylvie.marguet@cea.fr, christian.gollwitzer@ptb.de*

This is the Supplementary Information (SI) to the paper describing CDEF, a software library to compute scattering form factors for arbitrarily shaped particles. Among other information, the SI contains additional simulated and fitted scattering curves. The chapters in this SI correspond directly to the chapters with the same number in the main manuscript.

S1. Implementation details of CDEF

S1.1. CDEF vs. the SPONGE vs. other methods

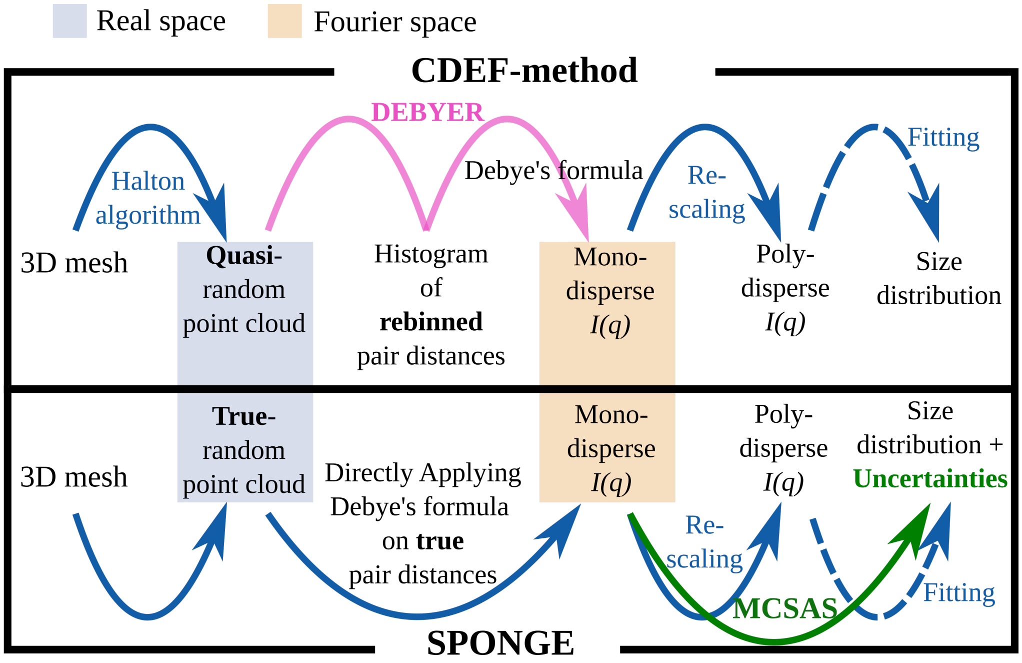

Fig. S1. Comparison between CDEF and the SPONGE. Both methods use Debye's scattering formula to calculate the single-particle scattering profile starting from the associated three-dimensional (3D) mesh which represents the arbitrary particle shape.

In this section, CDEF is compared to similar pre-existing approaches for the computation of form factors using Debye's scattering formula.

The SPONGE has already proven to successfully simulate helicoidal supramolecular copolymers at different structural parameters (Aratsu *et al.*, 2020), therefore it is used here to verify the accuracy of the much faster CDEF in order to use the latter with trust. In contrast to the SPONGE, which directly evaluates Debye's formula for each q, Debyer reduces the computational effort by a two-step process where the binned distance distribution histogram is first computed and subsequently reused for each value of q. This approximation should have a negligible effect if the bin size of the

histogram is sufficiently small compared to the particle size.

By default, CDEF also employs a quasi-random filling as opposed to the true random filling as used by the SPONGE. As a result, a smaller number of punctiform scatterers can be used w.r.t. the same usable q-range which also reduces computational effort.

All in all, the basic concept of CDEF originates from (Hansen, 1990) as well as (Pedersen *et al.*, 2012). For instance, Hansen calculated $I(q)$ of a complex-shaped fibrinogen using the distance distribution function of a corresponding quasi-random point cloud (Hansen, 1990), without rebinning the distance distribution however. Moreover, Hansen focused on calculating single-particle $I(q)$, but did not consider polydisperse particle ensemble. Pedersen et al., on the other hand, evaluated polydisperse immune-stimulating-complex particles by applying Debye's equation. In doing so, the true-random particle cloud including 40 000 scatterers was split into 10 subsets of 4 000 scatterers from which 10 single pair distance histograms were calculated and then summed up to generate the total histogram (Pedersen *et al.*, 2012). Compared to the direct computation of the total histogram, this procedure lowers computational cost by one order of magnitude (Pedersen *et al.*, 2012), but at the expense of accuracy.

Even though the approach of Pedersen *et al.* (2012) is very similar to CDEF, corresponding software has not been published to our knowledge such that it would be accessible to SAXS experimentalists.

Since CDEF and SPONGE use a random filling method (i.e., quasi-random or true random) instead of the exact atomic positions of the periodic lattice of particles, the computational time for both methods is significantly less than for a periodic lattice. For the evaluation of Au nanocubes with face-to-face distance 50 nm, for instance, choosing 30 000 rather than $\sim 5.2 \cdot 10^6$ scatterers which is the approximate number of atoms in a gold crystallite of this size (Pauling, 1947), decreases computational effort by four orders of magnitude ($\sim$ 30 500). The computation of a single SAXS profile

using CDEF with 30 000 scatterers requires $\sim$ 500 ms on a single modern desktop computer with a quad core processor. Hence, this would need more than 4 hours in case of $5.2 \cdot 10^6$ scatterers, which justifies the simplification using the quasi-random filling instead of the exact crystallographic positions.

In addition, CDEF allows to individually adjust the scattering length of each single scatterer as described by Pedersen *et al.* (2012) leading to much more versatile applications such as fitting core-shell-structured particles.

Besides the use of a periodic grid for particle formation, the use of a Poisson disc algorithm would also be conceivable, but it converges slower and slower with increasing scattering point number than the use of a random distribution method. Figure S2 shows here that the Poisson disc method is not closer to the analytical solution.

For SAXS on isotropic macromolecules, similar approaches have been made in the past by the European Molecular Biology Laboratory (EMBL) to calculate $I(q)$ from the scattering properties of the underlying substructure by introducing CRYSOL, which are part of the ATSAS software package (Franke *et al.*, 2017; Svergun *et al.*, 1995). However, unlike CDEF, CRYSOL calculates $I(q)$ of macromolecules in dilute solution from the *exact* crystallographic positions and form factors of their individual atoms (Svergun *et al.*, 1995). This involves spherical averaging using spherical harmonics and their orthogonality properties to obtain a simplified expression for the total molecular form factor. Since CRYSOL also accounts for scattering from the missing water molecules and the hydration shell of the molecules as correction terms for the total form factor of the molecules, it can resolve scattering profiles up to $q \leq 4\,\mathrm{nm}^{-1}$ (Svergun *et al.*, 1995). However, despite the higher q range, it is not necessary to use accurate crystallographic positions to adequately calculate $I(q)$ of suspended nanoparticles. Moreover, the electron contrast of particles composed of metals or oxides is much higher than that of biomolecules, which allows us to ignore a possible hydration shell

for accurate SAXS data evaluation. Another software from ATSAS called DAMMIN calculates scattering patterns based on a substructure consisting of artificial scatterers called dummy atoms, which is generally more similar to CDEF than CRYSOL (Svergun, 1999; Franke *et al.*, 2017).

Lastly, there is the comprehensive software package DEBUSSY using Debye's formula which offers users a GUI and therefore allows one to compute scattering pattern of crystalline or non-ordered nanostructures without any programming knowledge (Cervellino *et al.*, 2015). Even though users of DEBUSSY are able to generate nano-scale clusters, such as nanoparticles or quantum dots, by defining the coordinates of the underlying (atomic) structure to calculate the corresponding SAXS scattering profiles (Bertolotti *et al.*, 2016), CDEF overall seems to be more suited to analyze SAXS scattering pattern of nanoparticles in the upper nano-scale region due to the much easier cloud building feature of simply loading an stl-file defining the particle's shape and then choosing the desired filling algorithm.

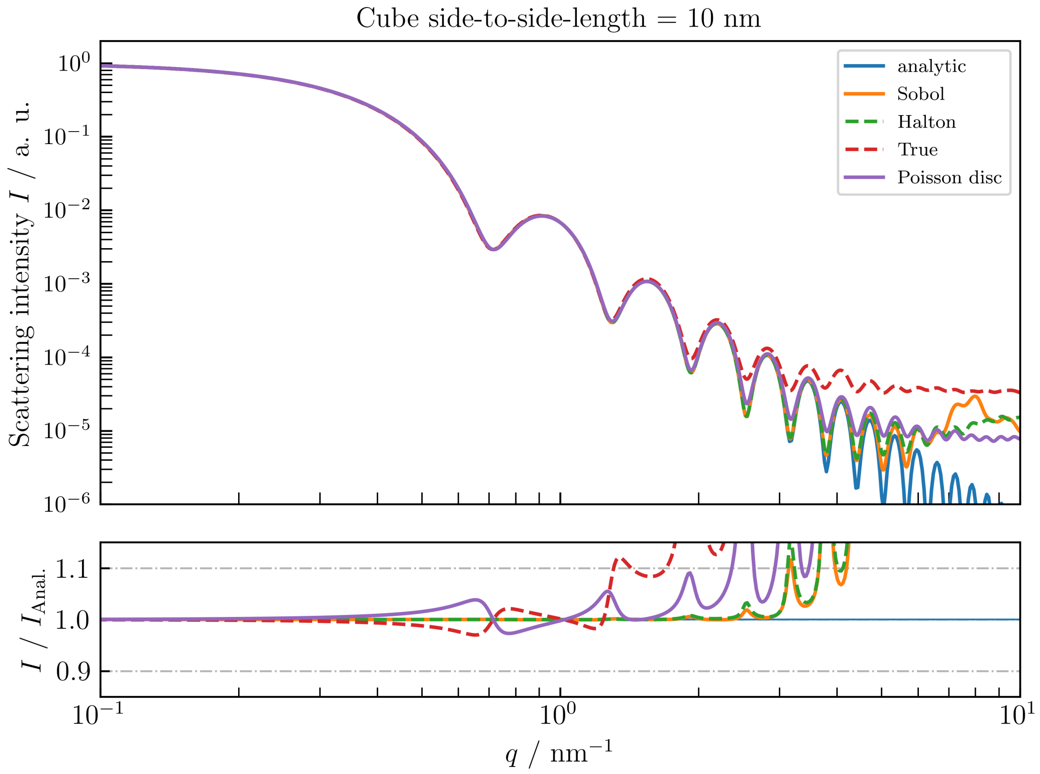

Fig. S2. Comparison of normalized single-particle SAXS profiles using CDEF with the exact analytic SAXS profile $I_{\text{Anal.}}$ of a cube with edge length $= 10\,\text{nm}$ and electron contrast $\Delta\rho = 1\,\text{nm}^{-3}$. All point clouds consist of $\sim 30\,000$ scatterers. Using a Poisson-disc-distribution does not result in a higher agreement of the corresponding scattering profile with the analytic profile when compared to the Sobol or Halton distribution.

S2. CDEF vs. analytic formulae

This section is intended as a supplement to the comparison between numerically calculated and analytical form factors. Here we also present background modeled scattering curves by changing the number of filled bins of the corresponding pair distance distribution.

For the true random distribution, it is generally recommended to exclude only the self-correlation as suggested by Pedersen *et al.* (2012), which correpsonds to the first histogram bin with zero distance. For the quasi-random case, users of CDEF have several options. According to the comparisons shown below, the best correspondence in the mid q range is achieved by not deleting any bins. However, if this leads to an excessive background for higher q values and the number of scatterers cannot be increased, a few histogram bins can be deleted, starting with the second one, and leave the first bin untouched. In this chapter, we show examples for the different particle shapes of how many consecutive bins need to be deleted for the curves to be closest to the analytical solution.

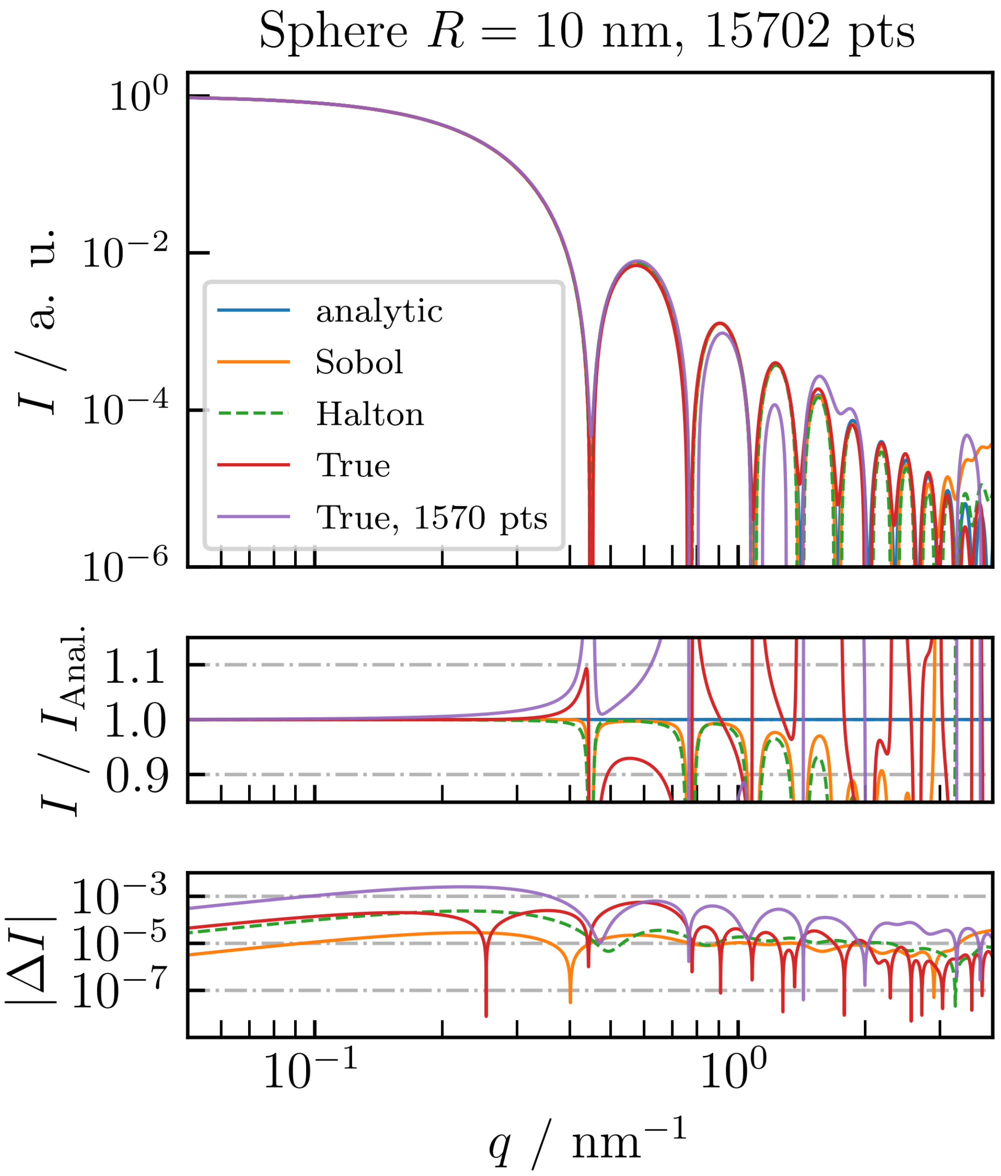

Fig. S3. Comparison of normalized single-particle SAXS profiles using CDEF with the exact analytic SAXS profile $I_{\text{Anal.}}$ of a sphere with radius $R = 10\,\text{nm}$ and electron contrast $\Delta\rho = 1\,\text{nm}^{-3}$. Manual changes to the pair distance histogram allow user of CDEF to increase the usable q-range of the numeric curves through *modeling of the artificial background signal*.

For proper modeling of the artificial background signal of the quasi-random SAXS

pattern of the spherical cloud, the first 60 of 10 000 bins were deleted ($n_{k=1,\ldots,60} = 0$), except the first bin $n_{k=0}$ to avoid excessive background modeling. The background modeled SAXS profiles of the quasi- and true-random ($n_{k=0} = 0$) filling algorithms are quite similar with a small advantage for the quasi-random distributions in the lower and middle q-region w. r. t. ($I\,/\,I_{\mathrm{Anal.}}$)-plot (fig. S3). Interestingly, this remains the case even if the background modeled true-random curve is compared to the non-modeled quasi-random profiles (fig. S4).

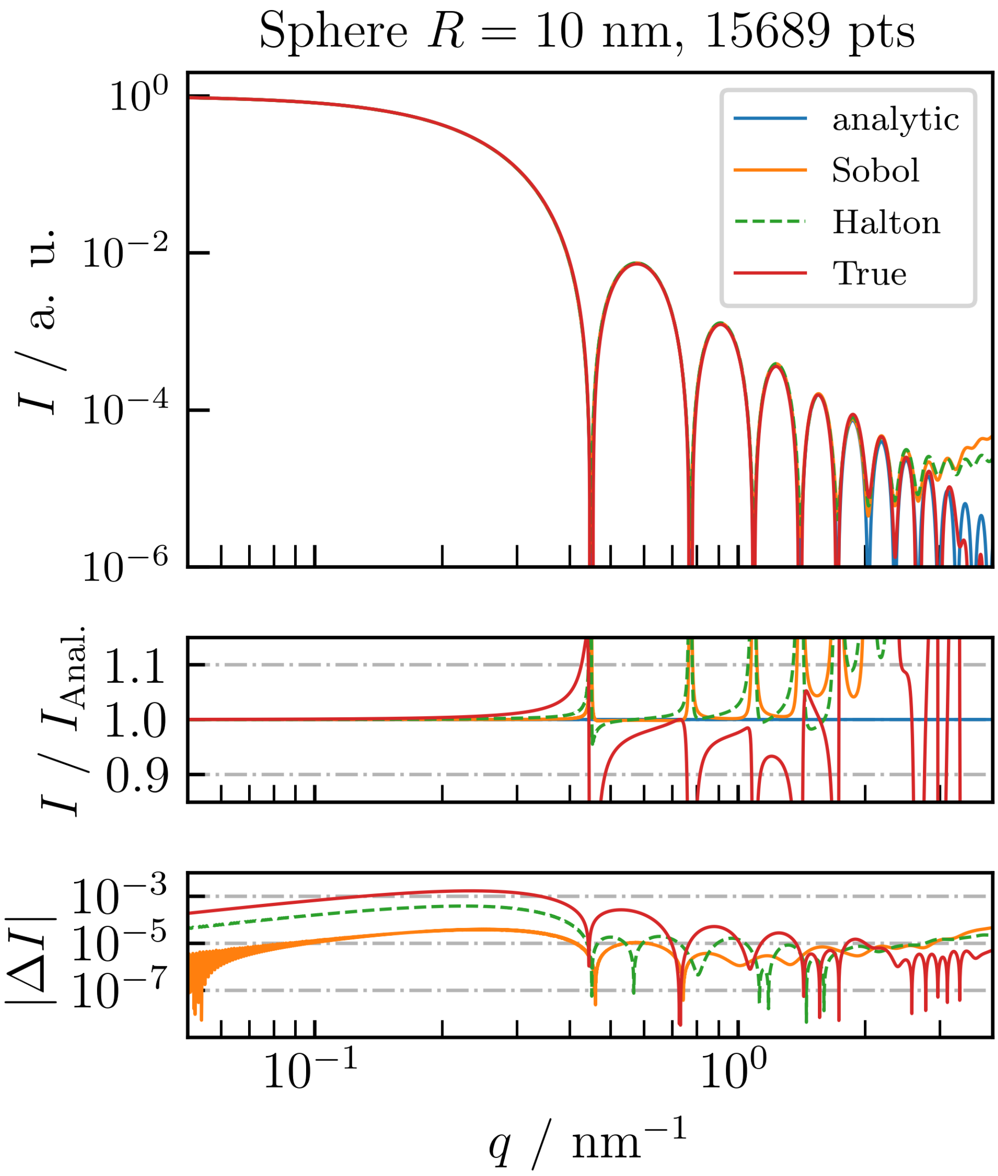

Fig. S4. Comparison of normalized single-particle SAXS profiles using CDEF with the exact analytic SAXS profile $I_{\mathrm{Anal.}}$ of a sphere with radius $R = 10\,\mathrm{nm}$ and electron contrast $\Delta\rho = 1\,\mathrm{nm}^{-3}$. Only the true-random profile is background modeled ($n_{k=0} = 0$).

In order to evaluate the performance of CDEF for particles with lower symmetry, fig. S5 compares analytically and numerically obtained single-particle SAXS profiles of a cylinder with aspect ratio $L\,/\,R = 6$ and $\Delta\rho = 1\,\mathrm{nm}^{-3}$. The analytic SAXS curves were calculated using the well-known expression (Guinier & Fournet, 1955; Galantini *et al.*, 2004). For the calculation of each numeric profile, cylindrical clouds of $N \approx 30\,000\,\pi/4 \approx 23\,560$ scattering points were generated similarly to the spherical clouds before. The quasi-random profiles match the analytic one up to the 4th local maximum, whereas the true-random pattern hardly matches the analytic solution up to the first local maximum.

After deletion of the first bin ($n_{k=0} = 0$) of the true-random histogram as well as 35 out of 10 000 ($n_{k=1,\ldots,35} = 0$) bins of the quasi-random histogram, the quasi-random SAXS profiles are still more consistent with the analytic solution regarding the lower and middle q-range (fig. S6). The specific upper bin limit of 35 for the cylinder was determined by gradually clearing bins until an adequate background modeling was obtained, as done for the spherical and the cubic point cloud. As for the spherical cloud, comparing the background modeled true-random curve to the non-modeled quasi-random profiles indicates that the quasi-random distribution seems to be superior in this q-region (fig. S5 vs. S6).

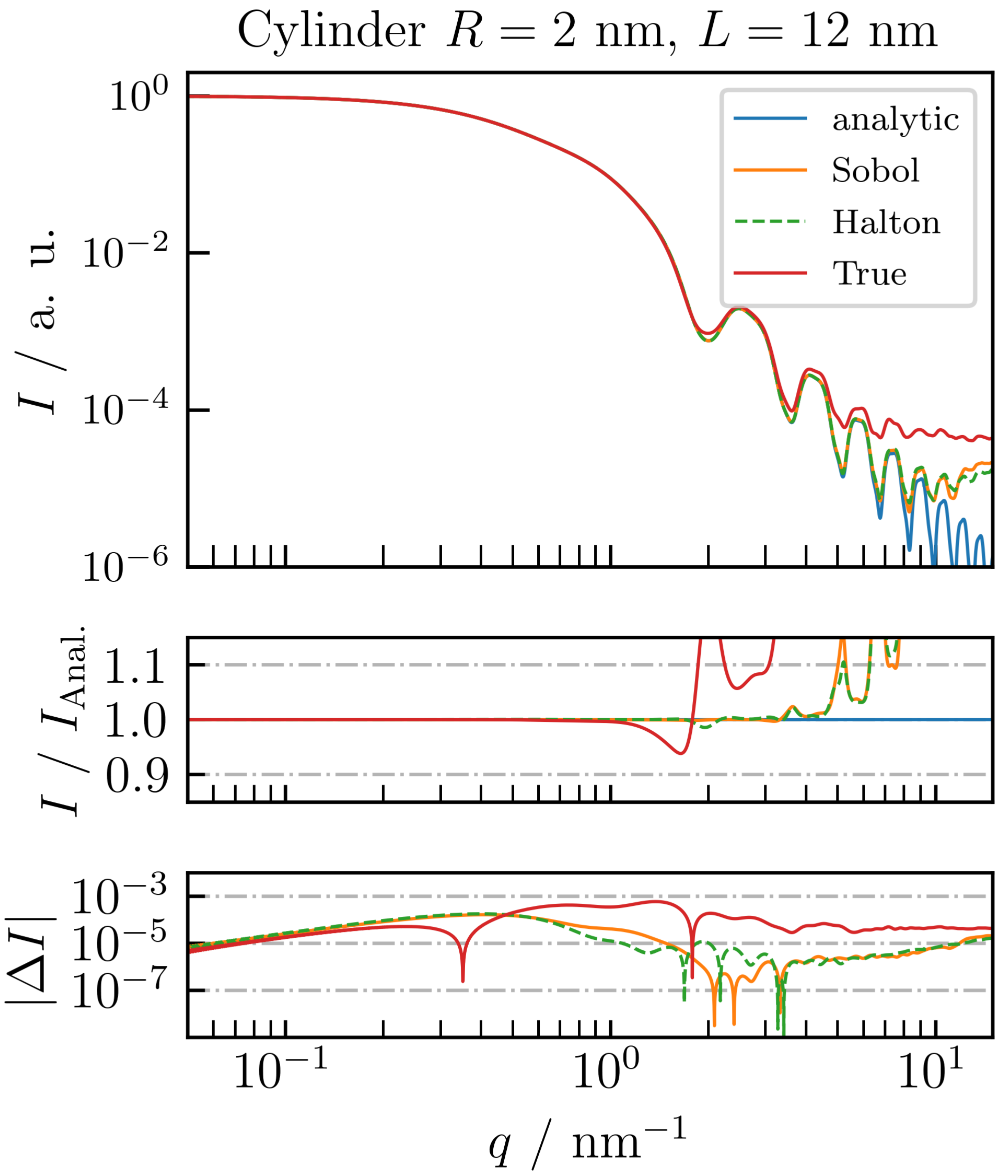

Fig. S5. Comparison between numeric and analytic single-particle SAXS profiles of a cylindrical particle with radius $R = 2\,\mathrm{nm}$, length $L = 12\,\mathrm{nm}$ and electron contrast $\Delta\rho = 1\,\mathrm{nm}^{-3}$.

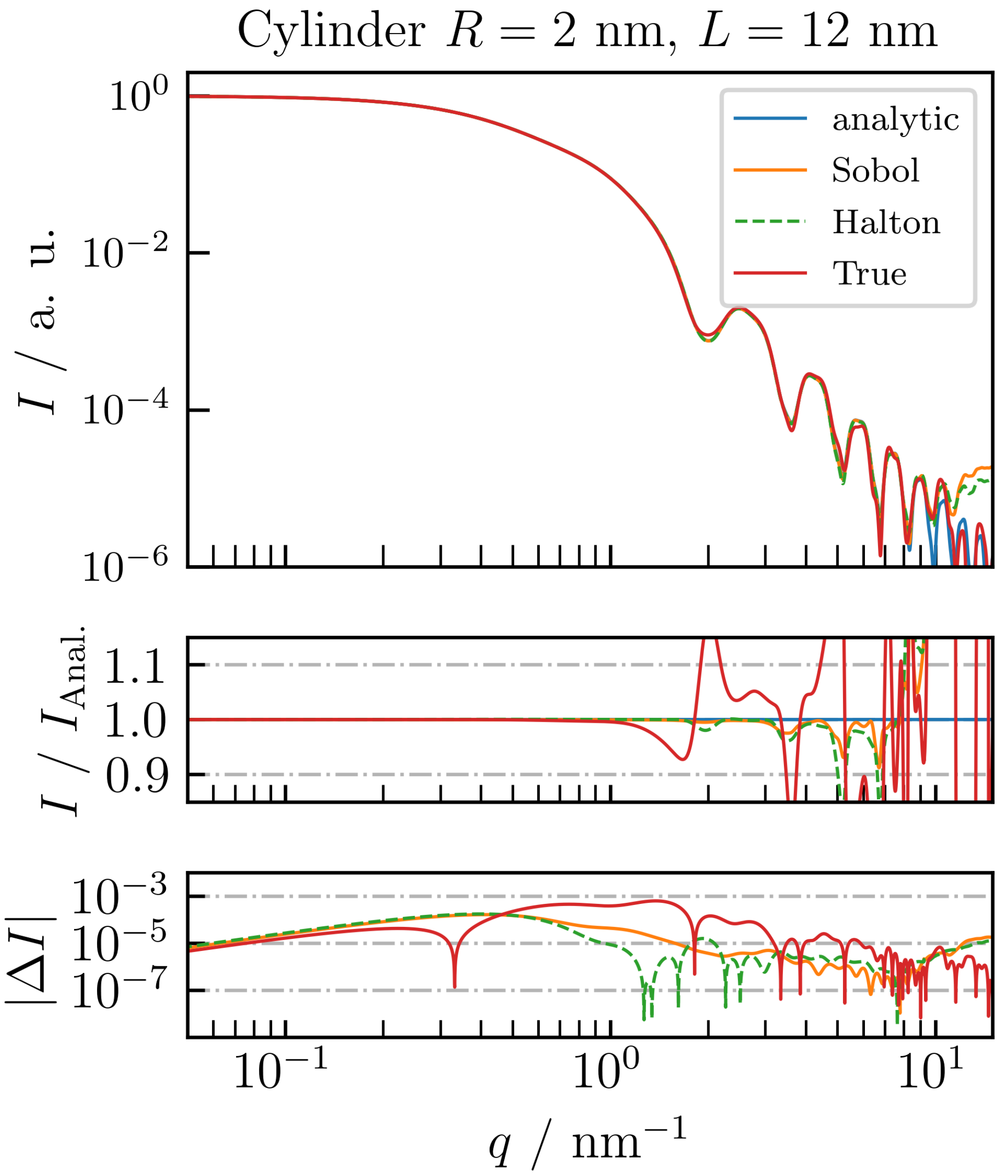

Fig. S6. Comparison between numeric and analytic single-particle SAXS profiles of a cylindrical particle with radius $R = 2\,\mathrm{nm}$, length $L = 12\,\mathrm{nm}$. All profiles are background modeled.

Regarding ideal cubes, CDEF is also able to adequately match $I_{\mathrm{Anal.}}$ as depicted in fig. S7. The analytic curve was calculated by averaging the expression from (Mittelbach & Porod, 1961) over two independent spatial coordinates. For this particular cube with side-to-side length 10 nm, CDEF with 30 000 scatterers as well as $I_{\mathrm{Anal.}}$ were evaluated on 999 q-values, whereas for the calculation of each numeric profile CDEF needed only $\sim$ 500 ms. Background modeling was conducted by clearing the first 45 of 10 000 bins ($n_{k=1,\ldots,45} = 0$) for the quasi-random profiles as well as the first bin ($n_{k=0} = 0$) for the true-random profile. This specific upper bin limit of 45 for the cube was determined by gradually clearing bins until an adequate background modeling was obtained, as done for the spherical and the cylindrical point cloud.

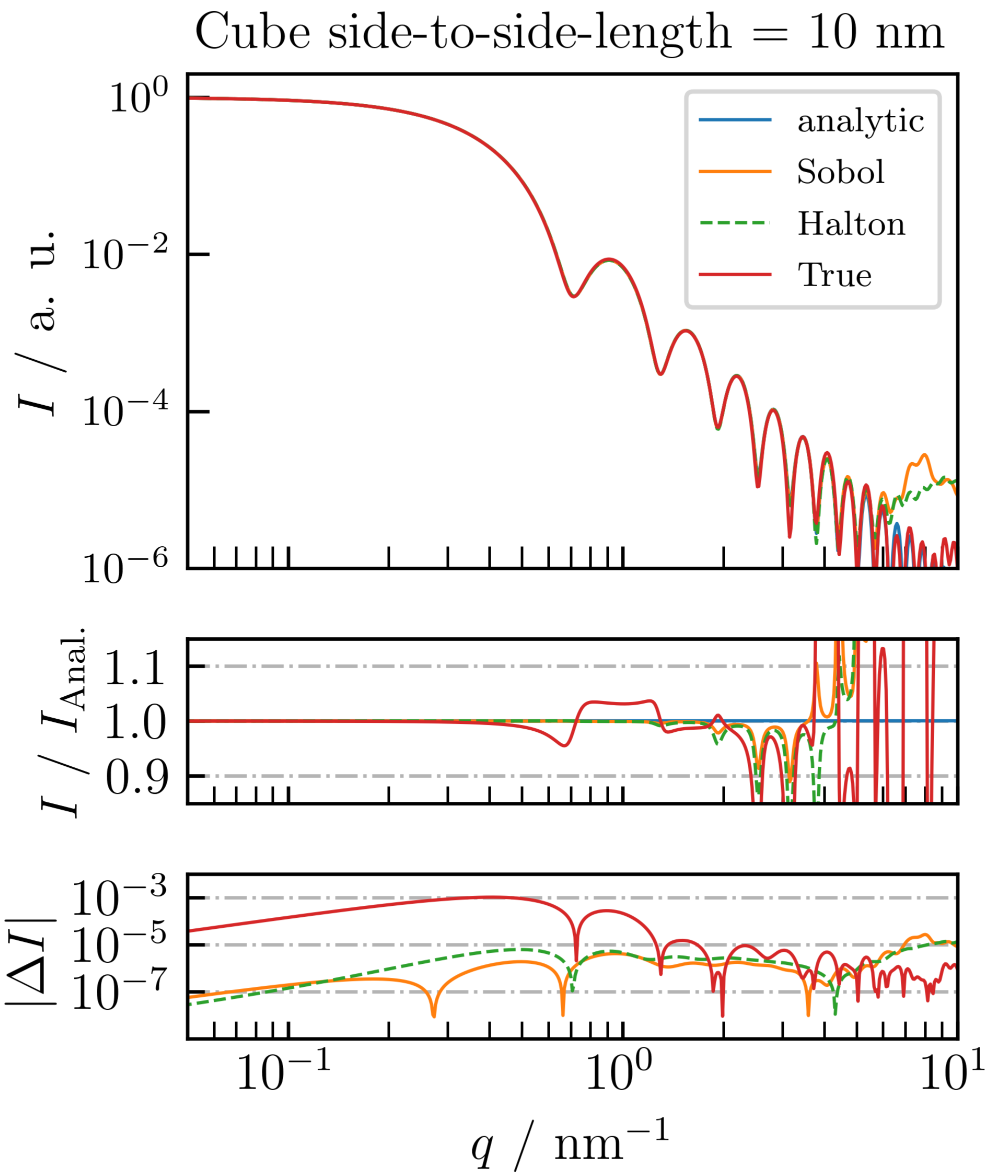

Fig. S7. Comparison between numeric and analytic background modeled SAXS profiles of an ideal cube with side-to-side length 10 nm and electron contrast $\Delta\rho = 1\,\text{nm}^{-3}$. In the lower and middle q-region, the quasi-random profiles are more consistent with the analytic profile compared the true-random one.

S3. Diverse models of cubic particles

This section describes the construction of the cubic shape with truncated edges.

S3.1. Hessian normal - cube with truncated edges

The Hessian normal form was used to parameterize the degree of truncation with respect to the cubic model with symmetrically cut edges. It describes the shortest distance D of a point with the Euclidean position $\vec{x}$ relative to a given plane described by a support vector $\vec{a}$ and normal vector $\vec{n}$:

$$D = (\vec{x} - \vec{a}) \cdot \vec{n}. \tag{1}$$

For each scattering point with position $\vec{x}$, D is calculated for each of the 12 sectional planes which cut the edges of the cubic model. If $D < 0$ for all 12 sectional planes, the point is located inside the cube, because the defined normal vectors $\vec{n}$ are pointing away from the cloud's center by definition. All outside points are deleted by setting their corresponding form factor to zero. The degree of truncation can be influenced by equally varying the length of all $\vec{a}$ by modifying an introduced truncation factor T, with

$$\vec{a} = T \frac{L}{\sqrt{2}} \vec{n}. \tag{2}$$

With this definition, $T = 1$ indicates no truncation and corresponds to the *ideal* cube.

S4. Results and Discussion

With this chapter we want to complement the chapter of the published work, where only the results of the cubic model with rounded edges are presented. In addition, this chapter contains the fits with an ideal cube as well as a cube with truncated edges. First, the results for the ideal cube are shown, followed by the truncated cube.

For comparison, a spherical model was additionally included in the evaluation as depicted in fig. S8. The weak match between experimental data and spherical fit demonstrates the sensitivity of the scattering experiment to the particle shape. Even though the ideal cubic model also leads to pronounced deviations at $0.2\,\mathrm{nm}^{-1} < q$, in general it matches the oscillations with better agreement compared to the spherical model which is also confirmed by the corresponding values of χ^2. The spherical model only matches the Guinier region satisfyingly resulting in a mean diameter of $d = 65.5\,\mathrm{nm}$ with a standard deviation of $\sigma_\mathrm{d} = 6.7\,\mathrm{nm}$.

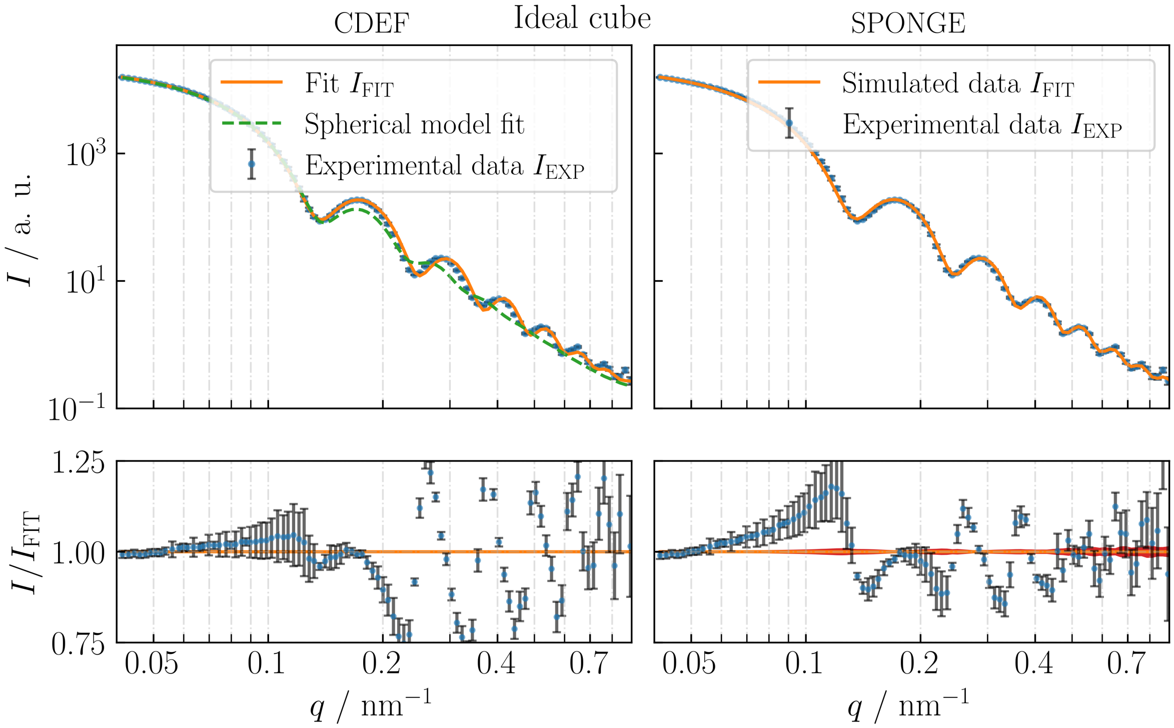

Fig. S8. CDEF versus the SPONGE. Fit results of Au nanocubes using the model of an ideal cube. Coupling of the SPONGE with MCSAS additionally reveals an uncertainty of I_{FIT} marked in red the SPONGE's I/I_{FIT}-Plot, thus an uncertainty of the underlying size distribution can be stated (fig. S9).

Using the ideal cubic model reveals a volume-weighted mean face-to-face-distance of $L = 52.5\,\mathrm{nm}$ with a distribution width of $\sigma_{\mathrm{L}} = 2.8\,\mathrm{nm}$. With the SPONGE we get an mean value of $L = (53.20 \pm 0.06)\,\mathrm{nm}$ with a distribution width of $\sigma_{\mathrm{L}} = (2.7 \pm 1.0)\,\mathrm{nm}$ which leads to a relative deviation of $\Delta L\,/\,L \approx 1.5\,\%$ between CDEF and the SPONGE. Regarding the Guinier region, CDEF seems to give a slightly better fit compared to the SPONGE, whereas in the Porod region the SPONGE is superior.

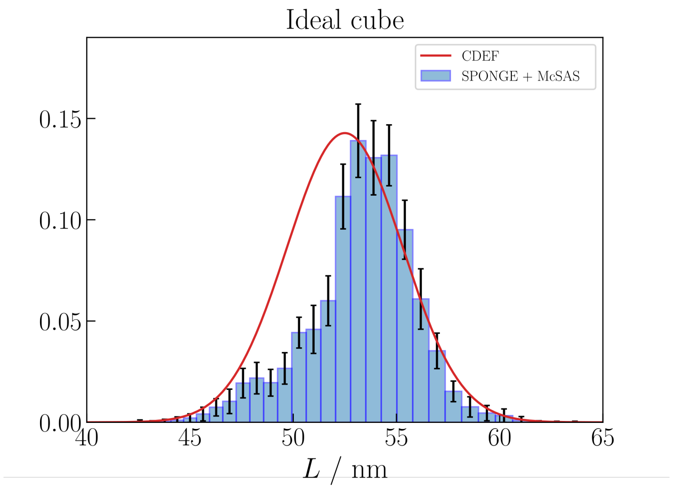

Fig. S9. CDEF vs. the SPONGE: Normalized distribution of side-to-side length of the Au nanocubes. The uncertainty of the volume-weighted distribution using the SPONGE with a mean value of $L = (53.20 \pm 0.06)$ nm is indicated by error bars. The volume-weighted distribution using CDEF reveals a mean value of $L = 52.5$ nm.

The truncated cubic model reveals a mean value of $L = 53.4$ nm with $\sigma_{\mathrm{L}} = 3.3$ nm, and a truncation factor of $T = 0.91$. From a geometrical perspective, this truncation factor means that on average ~ 2.6 nm are cut off on both sides of each edge. With the SPONGE we obtain a value of $L = (54.00 \pm 0.04)$ nm with a distribution width of $\sigma_{\mathrm{L}} = (3.0 \pm 0.8)$ nm is obtained. A reason for the relative deviation of $\Delta L\,/\,L \approx 1.1\,\%$ between CDEF and the SPONGE (as for the other cubic models) may result from the fact that CDEF is confined to a Gaussian size distribution, whereas the SPONGE is not. This assumption is also confirmed by the fact that there are no deviations in the corresponding single-particle and polydisperse scattering profiles when comparing CDEF with the SPONGE.

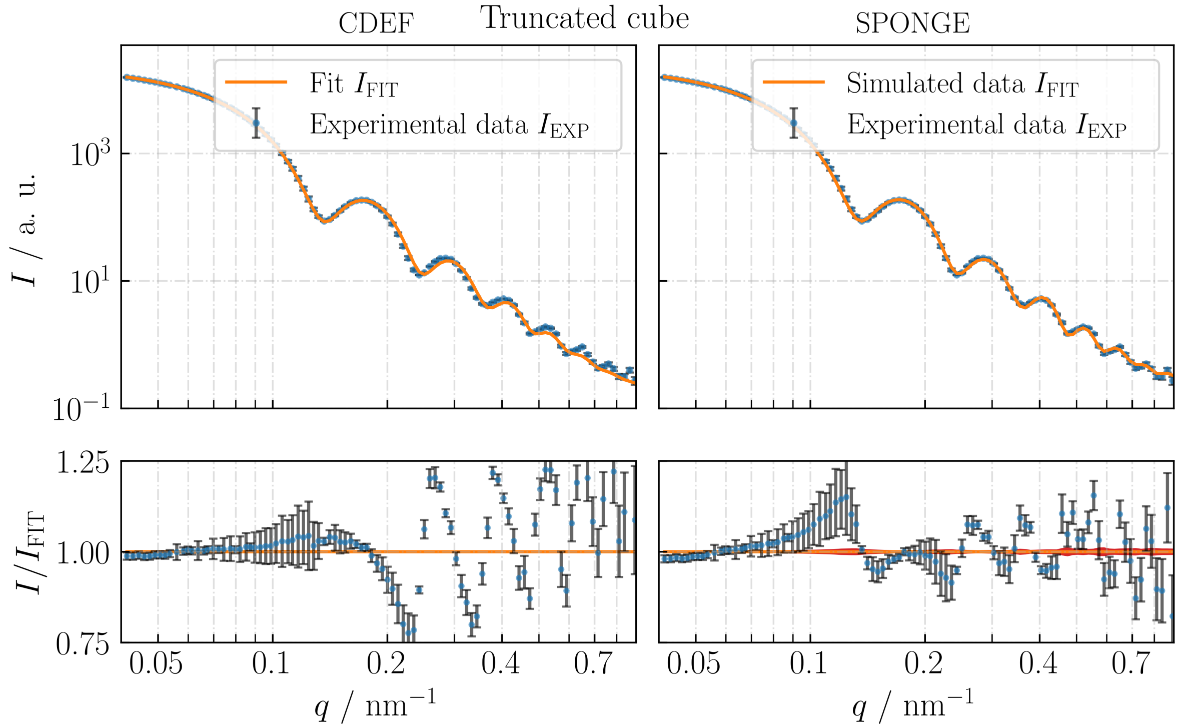

Fig. S10. CDEF versus the SPONGE. Fit results of Au nanocubes using a cubic model with truncated edges. Coupling of the SPONGE with MCSAS additionally reveals an uncertainty of I_{FIT} marked in red as explained in fig. S8, thus an uncertainty of the underlying size distribution can be stated (fig. S11).

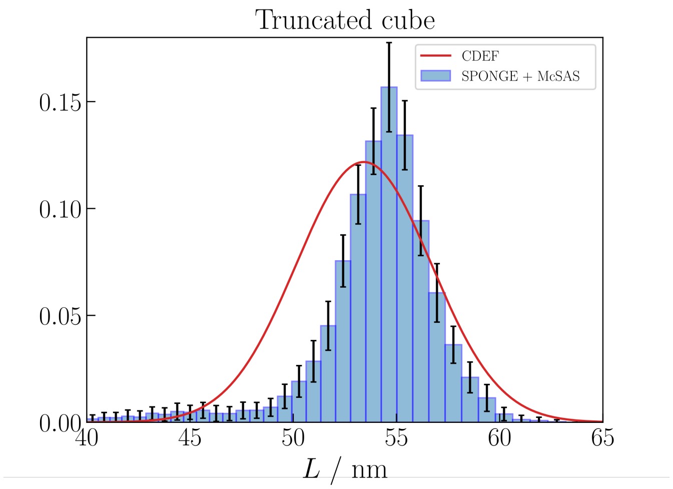

Fig. S11. CDEF vs. the SPONGE: the SPONGE's volume-weighted size distribution reveals a mean value of $L = (54.00 \pm 0.04)\,\text{nm}$. The volume-weighted distribution using CDEF shows an expectation value of $L = 53.4\,\text{nm}$.

References

Aratsu, K., Takeya, R., Pauw, B. R., Hollamby, M. J., Kitamoto, Y., Shimizu, N., Takagi, H., Haruki, R., Adachi, S.-i. & Yagai, S. (2020). *nature communications*, **11**(1), 1623.

Bertolotti, F., Dirin, D. N., Ibanez, M., Krumeich, F., Cervellino, A., Frison, R., Voznyy, O., Sargent, E. H., Kovalenko, M., Guagliardi, A. & Masciocchi, N. (2016). *NATURE MATERIALS*, **15**(9), 987–994.

Cervellino, A., Frison, R., Bertolotti, F. & Guagliardi, A. (2015). *Journal of Applied Crystallography*, **48**, 2026–2032.

Franke, D., Petoukhov, M. V., Konarev, P. V., Panjkovic, A., Tuukkanen, A., Mertens, H. D. T., Kikhney, A. G., Hajizadeh, N. R., Franklin, J. M., Jeffries, C. M. & Svergun, D. I. (2017). *Journal of Applied Crystallography*, **50**, 1212–1225.

Galantini, L., Giglio, E., Leonelli, A. & Pavel, N. V. (2004). *The Journal of Physical Chemistry B*, **108**, 3078–3085.

Guinier, A. & Fournet, G. (1955). *Small-Angle Scattering of X-rays*, chap. 2 - General Theory, 4 - Methods of Interpretation of Experimental Results. John Wiley & Son, Inc.

Hansen, S. (1990). *Journal of Applied Crystallography*, **23**, 344–346.

Mittelbach, P. & Porod, G. (1961). *Acta Phys. Austriaca*, **14**, 185–211.

Pauling, L. (1947). *Journal of the American Chemical Society*, **69**(3), 542–553.

Pedersen, J. S., Oliveira, C. L. P., Hübschmann, H. B., Arleth, L., Manniche, S., Kirkby, N. & Nielsen, H. M. (2012). *Biophysical Journal*, **102**, 2372–2380.

Svergun, D., Barberato, C. & Koch, M. H. J. (1995). *Journal of Applied Crystallography*, **28**, 768–773.

Svergun, D. I. (1999). *Biophysical Journal*, **76**, 2879–2886.

IUCr macros version 2.1.10: 2016/01/28
